# Characteristics of the Quality Improvement Content of Cardiac Catheterization Peer Reviews in the Veterans Affairs Clinical Assessment, Reporting, and Tracking Program

**DOI:** 10.1001/jamanetworkopen.2019.8393

**Published:** 2019-08-02

**Authors:** Jacob A. Doll, Mary E. Plomondon, Stephen W. Waldo

**Affiliations:** 1 Section of Cardiology, VA Puget Sound Health Care System, Seattle, Washington; 2 Division of Cardiology, Department of Medicine, Rocky Mountain Regional VA Medical Center, Aurora, Colorado

## Abstract

**Importance:**

Peer review is recommended for quality assessment in all cardiac catheterization programs, but, to our knowledge, the content of peer reviews and the potential for quality improvement has not been described.

**Objective:**

To characterize the quality improvement content of cardiac catheterization peer reviews.

**Design, Setting, and Participants:**

This quality improvement study used retrospective case review of diagnostic angiography and percutaneous coronary intervention procedures to characterize the major adverse event review process of the US Department of Veterans Affairs Clinical Assessment, Reporting, and Tracking (CART) program from January 1, 2012, to December 31, 2016. Data review and analysis took place from November 2017 to August 2018.

**Main Outcomes and Measures:**

Percentage of peer reviews reporting substandard care and opportunities for quality improvement.

**Results:**

A total of 196 643 diagnostic coronary angiograms and 62 576 percutaneous coronary interventions were performed in the Department of Veterans Affairs. Of these, 168 (0.1%) were triggered for review because of a self-reported major adverse event during the procedure. Of 152 cases with complete peer review data, care was adjudicated as not meeting the standard of care in 25 cases (16.4%). Concerns about operator judgment were identified in 46 cases (30.3%), about case selection in 26 (17.1%), about trainee supervision in 21 (13.8%), and about technical performance in 46 (30.3%). Reviewers made recommendations to improve operator performance in 63 cases (41.4%) and catheterization laboratory or hospital processes in 58 (38.2%).

**Conclusions and Relevance:**

While substandard care is infrequently identified in peer review of catheterization laboratory complications in the Department of Veterans Affairs, the process often generates recommendations for quality improvement. Peer review programs should focus on identifying quality improvement opportunities and providing meaningful feedback to operators.

## Key Points

**Question:**

Do peer reviews of cardiac catheterization laboratory complications provide useful content for quality improvement?

**Findings:**

In this quality improvement study, 152 cardiac catheterization cases selected for peer review because of the occurrence of a major adverse event were analyzed, and only 16.4% of cases were adjudicated as not meeting the standard of care. Concerns about operator performance and judgment were more common, and reviewers recommended improvements in operator performance and care processes in 41.4% and 38.2% of cases, respectively.

**Meaning:**

Peer review programs should focus on maximizing quality improvement opportunities even when the standard of care is met.

## Teaser

This quality improvement study characterizes the content of cardiac catheterization peer reviews in the US Department of Veterans Affairs (VA) Clinical Assessment, Reporting, and Tracking (CART) program.

## Introduction

Peer review is recommended for all cardiac catheterization programs as a mechanism to promote clinical proficiency and identify opportunities for quality improvement (QI).^1^ Review of cases with poor outcomes or unexpected complications (ie, morbidity and mortality conference) is a common form of peer review,^2,3^ but whether this activity yields content that may guide performance improvement has not been described, to our knowledge. In 2004, the US Department of Veterans Affairs (VA) launched the Clinical Assessment, Reporting, and Tracking (CART) program to study and address concerns about cardiovascular care quality.^4^ Since 2011, the VA CART program has convened a committee of interventional cardiologists to review major complications that arise during percutaneous coronary intervention (PCI). We sought to characterize the QI content of these peer reviews via a retrospective analysis of program records.

## Methods

We examined records from all peer-reviewed cases in the VA CART major adverse event (MAE) program from January 1, 2012, to December 31, 2016. Data review and analysis took place from November 2017 to August 2018. Cases were triggered for review by the in-laboratory occurrence of death, stroke, need for urgent coronary artery bypass graft surgery (CABG), or need for urgent unplanned PCI during diagnostic angiography (a trigger since 2015). Percutaneous coronary intervention operators reported the adverse event by selecting the complication from a templated list on the VA CART procedure report. Staff with the VA CART program compiled procedure reports and images from the diagnostic angiography or PCI procedure for dissemination to the MAE peer review committee. The peer review committee is composed of 8 to 12 VA interventional cardiologists, 2 of whom are designated to review each case and given access to the angiography files and all clinical documentation available on the VA national integrated electronic health record. The primary reviewer communicates with the operator to supplement the case documentation, and the secondary reviewer relies on documentation alone. The reviewers write an assessment of the procedure, including recommendations for improvement, using a standard questionnaire that solicits feedback on various aspects of the procedure (eAppendix in the Supplement). Each reviewer independently adjudicates the case using a 3-level rating system: level 1, indicating most experienced competent practitioners would have managed the case similarly in all aspects; level 2, indicating most experienced competent practitioners would have managed the case differently in 1 or more aspects; and level 3, indicating most experienced competent practitioners would have managed the case differently in several aspects. If either reviewer indicates a level 2 or 3 rating, the case proceeds to full committee review during a monthly teleconference. For full committee review, all members are provided access to the case documentation and angiography and invited to submit a level rating in advance of the teleconference. Committee discussion is focused on developing a consensus regarding the final rating and appropriate feedback to the operator. If consensus cannot be reached, a final rating is determined by majority vote. A report is issued to the operator, who can appeal the determination and address the committee personally on a subsequent teleconference. After additional discussion, a final report is issued to the operator and the hospital, including a final rating, a description of the committee discussion, and recommendations for improvement.

In this study, individual reviewer reports and the committee summary report (when present) were reviewed for each case. We described the frequency and indications of MAE cases, reviewer ratings, and content of feedback with summary statistics. To assess the completeness of event capture, we subsequently identified all deaths that occurred on the same day as a diagnostic catheterization or PCI procedure but were not reported to VA CART. We reviewed relevant clinical documentation to understand the cause of death and association with the catheterization procedure. This review was considered a QI activity within the scope of VA CART program operations and not subject to review by the institutional review board. This report adheres to the Revised Standards for Quality Improvement Reporting Excellence (SQUIRE) reporting guideline.

## Results

Overall, 196 643 diagnostic coronary angiograms and 62 576 PCIs were performed in the VA from January 1, 2012, to December 31, 2016. Of these, 168 (0.1%) were triggered for review by the VA CART program. Full documentation was available for review of 152 cases, including 78 deaths (51.3%), 47 strokes (30.9%), 24 emergent CABG surgical procedures (15.8%), and 3 unplanned PCIs during diagnostic angiography (2.0%). Documentation for the remaining 16 cases (5 deaths [31.3%], 6 strokes [37.5%], 1 emergent CABG surgical procedure [6.3%], and 4 unplanned PCIs [25.0%]) was irrevocably lost in a software failure. After review, 127 cases (83.6%) were rated level 1, 19 (12.5%) were level 2, and 6 (3.9%) were level 3 (ie, care was adjudicated as not meeting the standard of care in 25 cases [16.4%]). All cases rated level 3 were deaths. Reviewers were concordant in 121 cases (79.6%), but 31 cases (20.4%) were discordant and required full committee review to determine a final rating (Table). The primary reviewer (who spoke with the operator) was more likely to give a level 1 rating than the secondary reviewer (126 cases [82.9%] vs 116 cases [76.3%]).

**Table.** Comparison of Reviewer 1 and Reviewer 2 Peer Review Ratings in 152 Cases

Reviewer 2 Rating	No.			
	Reviewer 1 Rating			Total
	Level 1	Level 2	Level 3	
Level 1	108	7	1	116
Level 2	14	11	1	26
Level 3	4	4	2	10
Total	126	22	4	152

Concerns about operator judgment were identified in 46 cases (30.3%), about case selection in 26 (17.1%), about trainee supervision in 21 (13.8%), and about technical performance in 46 (30.3%). Reviewers identified potential for improvement in catheterization laboratory processes and operator performance in 58 cases (38.2%) and 63 cases (41.4%), respectively.

Feedback to the operators, when provided, generally focused on the following: (1) case selection, including the indication for revascularization and appropriate discussion of CABG surgery candidacy prior to PCI; (2) preprocedural planning, including need for hemodynamic support or atherectomy; and (3) procedural technique, including use of devices and adjunctive antithrombotic medications. In 2 cases (1.3%), the committee recommended a more extensive evaluation of PCI operator and catheterization laboratory quality.

An additional 107 deaths occurred on the same day as diagnostic coronary angiography or PCI during the study period but were not reported to VA CART. Of these, 10 (9.4%) were in-laboratory deaths and should have been reported by the operator to the MAE program. An additional 66 deaths (61.7%) were considered potentially associated with the cardiac procedures, and 31 deaths (28.9%) were considered unrelated to the procedure.

## Discussion

These data describe the processes of the only national peer review program for cardiac catheterization laboratories, functioning in the largest integrated health care system in the United States. Overall, peer review programs generally target 2 related, but sometimes competing, priorities: (1) quality assurance and (2) QI.^5^ Quality assurance activities are designed to assess and maintain care standards, with a focus on identifying and correcting outlier performance. In contrast, QI activities avoid negative attribution while targeting opportunities for systems change and education. In the VA CART program, the quality assurance function of peer review is infrequently needed. Only 25 cases (16.4%) over 5 years were determined to not meet the standard of care, and concerns about physician competency were very rare. This is similar to a single-center report of morbidity and mortality activities for PCI,^2^ in which physician performance was judged “very controversial or unacceptable” in only 5.9% of reviewed cases.

However, the VA CART MAE peer review program often generated QI content even when the standard of care was met. More than 40% of cases prompted recommendations for improved operator performance, and feedback regarding case selection, procedural planning, and technical performance was common. Despite the ubiquity of morbidity and mortality and case review conferences in US catheterization laboratories, there has been little research to describe successful peer review programs. Prior reports of peer review processes for PCI have focused on structural aspects of peer review and have not examined the QI output of the programs.^2,3^ For example, a 2017 report described review of 157 PCI cases over 10 years at a single academic center.^2^ Cases were triggered for review by the occurrence of at least 1 of 9 clinical triggers entered into the clinical record by catheterization laboratory staff. Cases were reviewed monthly by faculty using a standardized scoring system. The VA MAE program uses many of the same structural elements as this program but applies them on a national level, using anonymous reviews from clinical experts. Further research is required to determine the strengths and limitations of local vs national systems for peer review. The program assesses only MAEs, and it is unknown if more frequent random case review (as recommended by clinical societies^5^) or review of less serious complications would yield similar QI content. Complications were self-reported by the operator, and therefore may be subject to underreporting, although only 10 in-laboratory deaths were unreported over a 5-year period. In addition, we only captured complications occurring in the catheterization laboratory. This resulted in complication rates that are low compared with other health systems.^2,6^ A review of deaths occurring on the same day of the procedure but after the patient was transferred out of the catheterization laboratory revealed that most were potentially associated with care received in the laboratory. We are therefore exploring alternative mechanisms to identify poor procedural outcomes and improve capture of MAE.

### Limitations

This study has several important limitations. We cannot determine the effect of this program on overall cardiac catheterization outcomes at the VA, although peer review is an important component of a unique quality framework that may contribute to improved PCI outcomes at lower cost compared with non-VA facilities.^7^ Despite progressively increasing preprocedural risk, outcomes of patients treated with PCI at VA hospitals remained constant from 2009 to 2015, a period that spans the initiation of the VA CART MAE program in 2011.^8^ However, a randomized clinical trial may be required to determine the effect of peer review of clinical outcomes. It is also unclear if this VA experience is generalizable to other settings. The VA is a large national network of hospitals and physicians, all connected through a common electronic health record. Other health systems may require alternative solutions to monitor and improve catheterization laboratory quality.

## Conclusions

Peer review of catheterization laboratory complications serves an important quality assurance and QI role in the VA. Opportunities for improvement are often identified even when the standard of care is met. Peer review programs should focus on identifying QI opportunities and providing meaningful feedback to operators.

## Notes

Supplement. **eAppendix.** Templated Peer Review Form for the VA CART MAE Program
