# Supplementary material for: Characteristics of the Quality Improvement Content of Cardiac Catheterization Peer Reviews in the Veterans Affairs Clinical Assessment, Reporting, and Tracking Program
Source: JAMA Netw Open. 2019 Aug 2;2(8):e198393. doi: 10.1001/jamanetworkopen.2019.8393 (PMC6681545; doi:10.1001/jamanetworkopen.2019.8393)
Supplement: Supplement. — eAppendix. Templated Peer Review Form for the VA CART MAE Program [file jamanetwopen-2-e198393-s001.pdf]

Supplementary Online Content

Doll JA, Plomondon ME, Waldo SW. Characteristics of the quality improvement content of cardiac catheterization peer reviews in the Veterans Affairs Clinical Assessment, Reporting, and Tracking program. *JAMA Netw Open*. 2019;2(8):e198393. doi:10.1001/jamanetworkopen.2019.8393

eAppendix. Templated Peer Review Form for the VA CART MAE Program

This supplementary material has been provided by the authors to give readers additional information about their work.

© 2019 Doll JA et al. *JAMA Network Open.*

eAppendix. Templated Peer Review Form for the VA CART MAE Program

Brief Summary of Clinical Course (free text):

Assessment (choose one):

- Expected MAE
- Unexpected MAE

<u>Assessment Details:</u>

Opportunities identified for system or process improvements?

- Yes (if yes, please explain)
- No

Summary Assessment of Care: Clinical Judgement

- No Concern
- Minor Concern
- Major Concern

Summary Assessment of Care: Procedural Indication

- No Concern
- Minor Concern
- Major Concern

Summary Assessment of Care: Resident/Fellow Supervision

- No Concern
- Minor Concern
- Major Concern

Summary Assessment of Care: Technical Performance of the Procedure

- No Concern
- Minor Concern
- Major Concern

Final Assessment Level:

© 2019 Doll JA et al. *JAMA Network Open.*

- Level 1: Most Experienced, competent practitioners would have managed the case similarly in all aspects
- Level 2: Most experienced, competent practitioners would have managed the case differently in one or more aspects
- Level 3: most experienced, competent practitioners would have managed the case differently in several aspects.

Next Steps (free text):

© 2019 Doll JA et al. *JAMA Network Open.*
